# Tropical forests post-logging are a persistent net carbon source to the atmosphere

**DOI:** 10.1073/pnas.2214462120

**Published:** 2023-01-09

**Authors:** Maria B. Mills, Yadvinder Malhi, Robert M. Ewers, Lip Khoon Kho, Yit Arn Teh, Sabine Both, David F. R. P. Burslem, Noreen Majalap, Reuben Nilus, Walter Huaraca Huasco, Rudi Cruz, Milenka M. Pillco, Edgar C. Turner, Glen Reynolds, Terhi Riutta

**Affiliations:** ^a^School of Geography, Geology and the Environment, University of Leicester, Leicester LE1 7RH, United Kingdom; ^b^Georgina Mace Centre for the Living Planet, Department of Life Science, Imperial College London, Sunningdale SL5 7PY, United Kingdom; ^c^School of Geography and the Environment, University of Oxford, Oxford OX1 3QY, United Kingdom; ^d^Economic Planning Unit Sarawak, Sarawak, Kuching 93502E, Malaysia; ^e^School of Natural and Environmental Sciences, Newcastle University, Newcastle NE1 4LB, United Kingdom; ^f^School of Environmental and Rural Science, University of New England, Armidale NSW 2351, Australia; ^g^School of Biological Sciences, University of Aberdeen, Aberdeen AB24 2TZ, United Kingdom; ^h^Forest Research Centre, Sabah Forestry Department, Sandakan, Sabah 90175, Malaysia; ^i^Instituto de Ciencias de la Naturaleza, Territorio y Energías Renovables, Pontificia Universidad Católica del Peru, Lima 15088, Perú; ^j^Universidad Nacional de San Antonio Abad del Cusco, Cusco 08000, Perú; ^k^Department of Zoology, University of Cambridge, Cambridge CB2 3EJ, United Kingdom; ^l^South East Asia Rainforest Research Partnership, Danum Valley Field Centre, Lahad Datu, Sabah 91112, Malaysia; ^m^Geography Department, University of Exeter, Exeter EX4 4QE, United Kingdom

**Keywords:** carbon budget, logging, tropical ecology, carbon dynamics, land use

## Abstract

Logged and structurally degraded tropical forests are fast becoming one of the most prevalent land-use types throughout the tropics and are routinely assumed to be a net carbon sink because they experience rapid rates of tree regrowth. Yet this assumption is based on forest biomass inventories that record carbon stock recovery but fail to account for the simultaneous losses of carbon from soil and necromass. Here, we used forest plots and an eddy covariance tower to quantify and partition net ecosystem CO_2_ exchange in Malaysian Borneo, a region that is a hot spot for deforestation and forest degradation. Our data represent the complete carbon budget for tropical forests measured throughout a logging event and subsequent recovery and found that they constitute a substantial and persistent net carbon source. Consistent with existing literature, our study showed a significantly greater woody biomass gain across moderately and heavily logged forests compared with unlogged forests, but this was counteracted by much larger carbon losses from soil organic matter and deadwood in logged forests. We estimate an average carbon source of 1.75 ± 0.94 Mg C ha^−1^ yr^−1^ within moderately logged plots and 5.23 ± 1.23 Mg C ha^−^^1^ yr^−^^1^ in unsustainably logged and severely degraded plots, with emissions continuing at these rates for at least one-decade post-logging. Our data directly contradict the default assumption that recovering logged and degraded tropical forests are net carbon sinks, implying the amount of carbon being sequestered across the world’s tropical forests may be considerably lower than currently estimated.

## Significance

Logged tropical forests are counted as important carbon sinks in global carbon budgets due to the woody biomass they regain when they regrow following disturbance, but this assumption ignores the simultaneous carbon losses from the ecosystem. We found that, when quantifying all the source and sink terms of the ecosystem carbon budget, logged tropical forests are a net source of carbon to the atmosphere. This source persists at least 10 y following logging, meaning rates of carbon sequestration in recovering tropical forests are likely much lower than estimated.

One of the key ecosystem services that forests provide is the storage and sequestration of carbon (1). Tropical forests are particularly important within the global carbon budget, as they account for about 55% of global forest aboveground carbon stock (2) and approximately 40% of the global terrestrial carbon sink (3, 4). Despite this, tropical forest extent and functioning are threatened by climate change, land-use change, and structural degradation from logging, understory fires, and fragmentation (5). Logged tropical forests are now more widespread than unlogged forests in most areas of the tropics (6). Yet, there is a limited understanding of tropical forest carbon dynamics in response to logging. Studies that assess the impact of land-use change on carbon stocks and fluxes have mostly focused on deforestation (7–9), but it is estimated that total carbon losses from tropical forest structural degradation are similar to, or exceed, those from deforestation (10, 11). Tropical forest regrowth following disturbance, such as logging, can potentially provide an important carbon sink, as degraded areas regain biomass during recovery (12). To date, research into the recovery of logged and degraded forests has focused on the trajectory of biomass carbon stocks (2, 12–15), which is the “income” side of the carbon budget. But these studies do not serve as an assessment of the ecosystem carbon budget, as they do not estimate the “outgoings” of carbon losses from heterotrophic sources such as decomposition of deadwood and soil heterotrophic respiration, which was recently demonstrated to be elevated in logged relative to primary forests (16). Therefore, despite the higher tree growth rates in disturbed logged forests compared with unlogged forests (17), these systems may not function as net carbon sinks if past disturbances cause persistent carbon losses from soil and necromass stocks (16, 18).

Here, we present direct measurements of net ecosystem CO_2_ exchange and the complete carbon budget over a logging intensity gradient in a structurally degraded tropical forest landscape in Malaysian Borneo, a region which is a hotspot for deforestation and degradation (19). We used both of the two primary methods for quantifying CO_2_ exchange between the ecosystem and the atmosphere: eddy covariance and comprehensive biometric ground-based estimates and compared those with biometric estimates for nearby unlogged forests (16, 20). Both methods independently confirm that this landscape has been a substantial net carbon source to the atmosphere for at least a decade after logging.

## Results

### Eddy Covariance Estimates.

**Fig. 1.**
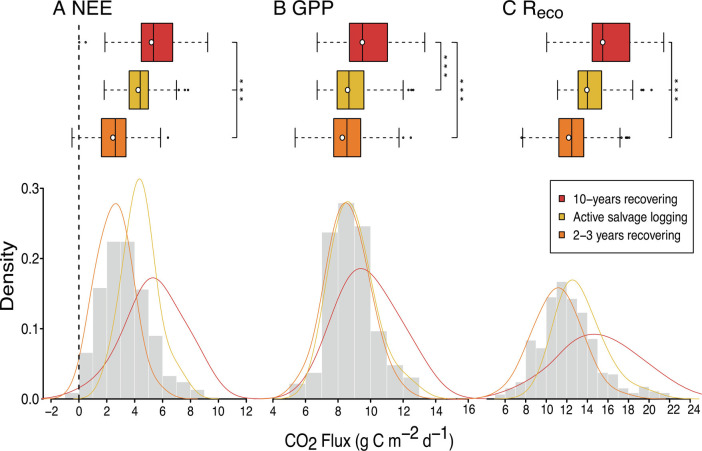
Net ecosystem CO_2_ exchange (*A*; NEE), gross primary productivity (*B*; GPP) and ecosystem respiration (*C*; R_eco_) estimated from an eddy covariance flux tower over a heavily logged forest within the SAFE project. Eddy covariance captured this site during three different measuring periods of: 10 y recovering since the previous round of logging (four times logged; 2012 to 2013; red), active salvage logging (2015; yellow), and 2 to 3 y recovery from salvage logging (2017 to 2018; orange). Boxplots and density lines show the range of the daily estimates in each period, with the white dots in the boxplots denoting the estimated mean value, with each day sampled as a replicate. Grey density histograms (bars) show the overall range for the site across all three measuring periods. Significant differences between periods, as determined by a Wilcoxon signed-rank test, are indicated with asterisks (*** indicating *P* < 0.05). Positive values indicate a net source of CO_2_ to the atmosphere. For all components see *SI Appendix*, Table S1 and *SI Appendix*, Fig. S1 for photographs of the landscape during the data collection periods.

The eddy covariance method found the ecosystem to be a net source of carbon to the atmosphere on 99% of 455 sampled days. Net ecosystem CO_2_ exchange, ecosystem respiration, and gross primary productivity were the lowest 2 to 3 y after salvage logging, highest ~10 y after the previous round of logging and intermediate during the active salvage logging period (Fig. 1 and *SI Appendix*, Table S1). Over all time periods, we observed an average net ecosystem CO_2_ exchange of 3.36 ± 1.76 g C m^−2^ d^−^^1^ (net source to the atmosphere). While the exact rate of net ecosystem CO_2_ exchange varied among the three periods (Fig. 1), this logged forest remained a net source of carbon throughout the 7-y observation period (Fig. 1). Net ecosystem CO_2_ exchange is the difference between gross primary productivity (incoming carbon) and ecosystem respiration (outgoing carbon). Both varied significantly among the three periods (Fig. 1), but gross primary productivity (mean across all sample periods: 8.84 ± 1.41 g C m^−^^2^ d^−^^1^) was consistently lower than ecosystem respiration (12.20 ± 2.9 g C m^−^^2^ d^−^^1^).

### Biometric Ground-Based Estimates.

**Fig. 2.**
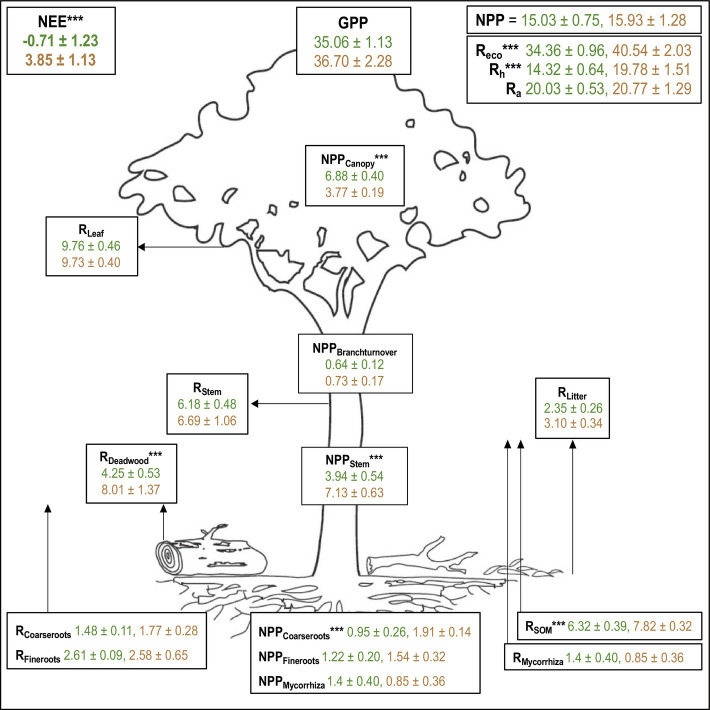
Components of the complete carbon budget (mean ± SE) for unlogged (reported in green, n = 6) and logged (reported in brown, n = 5) plots in Malaysian Borneo. Asterix (***) denotes a significant difference (*P* < 0.05, Wilcoxon rank-sum test) between logged and unlogged plots. For allocation of all components, see *SI Appendix*, Table S2. Units are Mg C ha^-1^ yr^-1^.

**Fig. 3.**
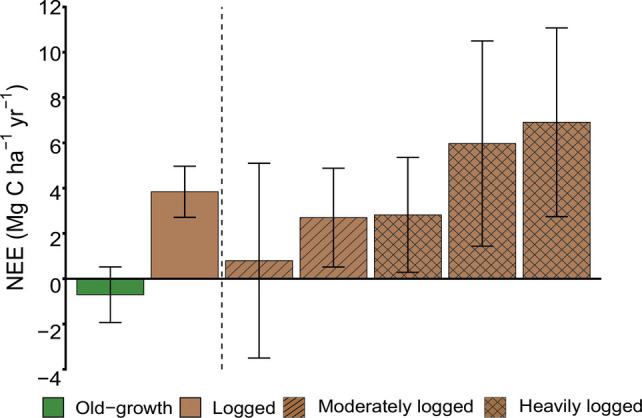
Net ecosystem CO_2_ exchange (NEE) estimated by biometric ground-based methods. Left of the dashed line show the mean (±1 SE) of six unlogged forest plots (green) and five logged plots (brown) with error bars representing variation across the plots. Right of the dashed line show the logged plots individually: two moderately logged plots (striped; *Left* to *Right*: SAF-03, SAF-04), and three heavily logged plots (hatched; *Left* to *Right*: SAF-01, SAF-02, SAF-05) with error bars representing within-plot uncertainty, estimated by propagation of SEs of the individually measured components of productivity and respiration. Positive values indicate a net source of CO_2_ to the atmosphere.

Plot-based biometric estimates were collected from 11 × 1-ha plots, which spanned a logging gradient from unlogged to heavily logged forest and included one plot located within the eddy covariance tower footprint. Such biometric estimates provide complete carbon budgets for both logged and unlogged forests (Fig. 2 and *SI Appendix*, Table S2) and show that unlogged plots were carbon neutral, with an average net ecosystem CO_2_ exchange of −0.71 ± 1.23 Mg C ha^−1^ yr^−1^. Logged plots, by contrast, had an average net ecosystem CO_2_ exchange of 3.85 ± 1.13 Mg C ha^−^^1^ yr^−^^1^ and were, therefore, a net source of carbon to the atmosphere. This difference between logged and unlogged plots was statistically significant [*t*(9) = −2.75, *P* = 0.015], although it obscures a high level of variability in net ecosystem CO_2_ exchange along the logging gradient (Fig. 3; plot-level estimates range from 0.80 to 6.91 Mg C ha^−^^1^ yr^−^^1^). This variation reflected the intensity of logging, with moderately logged plots having an average carbon loss roughly one-third that of heavily logged plots (Fig. 3).

There was no difference in total net primary productivity between logged and unlogged plots [Fig. 2; *t*(7) = −0.6, *P* = 0.56]. There was, however, an allocation shift away from the canopy and towards higher woody productivity in logged plots (20). Woody productivity was significantly greater in logged plots than in unlogged plots [*t*(8) = −3.86, *P* = 0.004], whereas canopy productivity was significantly smaller in logged plots than in unlogged plots [*t*(7) = 7, *P* = 0.0002]. There was no difference in gross primary productivity between logged and unlogged plots [Fig. 2; *t*(6) = −0.64, *P* = 0.93].

Logged plots exhibited significantly greater ecosystem respiration than unlogged plots [Fig. 2; *t*(6) = −2.5, *P* = 0.03]. This was caused by variation in heterotrophic respiration, which was significantly higher in logged than in unlogged plots [Fig. 2; *t*(5) = −3.31, *P* = 0.02]. Specifically, heterotrophic respiration from deadwood [*t*(5) = −2.56, *P* =0.049] and from soil organic matter [*t*(9) = −2.54, *P* = 0.032] was significantly greater in logged plots than in unlogged plots, while respiration from mycorrhiza [*t*(9) = 1, *P* = 0.33] and litter [*t*(9) = −1.74, *P* = 0.12] did not differ between the forest types. By contrast, there was no difference in total autotrophic respiration between logged and unlogged plots [*t*(6) = −0.53, *P* = 0.79], or in the individual components of autotrophic respiration including leaf [t(9) = 0.05, *P* = 0.96], fine root [W = 21, *P* = 0.32], coarse root [*t*(5) = −0.93, *P* = 0.66], and stem respiration [*t*(6) = −0.44, *P* = 0.68].

### Comparing Eddy Covariance and Biometric Ground-Based Estimates.

**Fig. 4.**
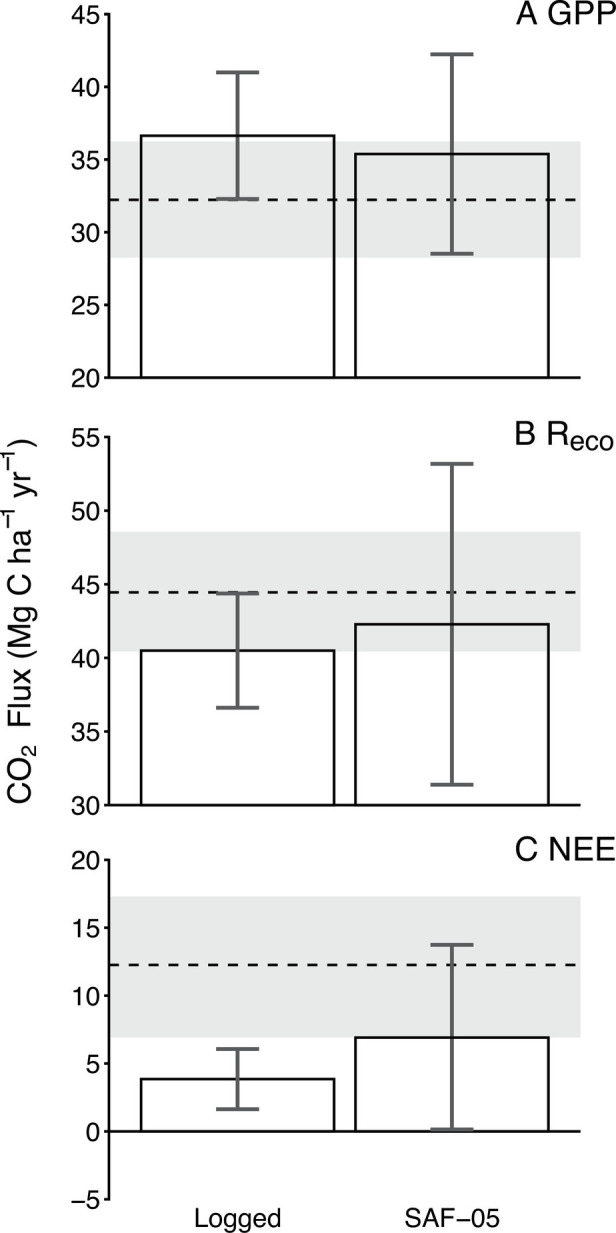
Comparison of the eddy covariance estimate (dashed line denotes the estimated mean and grey band the 95% CI, which incorporates the random uncertainty of eddy covariance) with the biometric estimates (bars ± 95% CIs) of logged forest mean values (n = 5 plots) and the SAF-05 plot, which is located within the eddy covariance tower footprint, for gross primary productivity (*A*; GPP), ecosystem respiration (*B*; R_eco_) and net ecosystem CO_2_ exchange (*C*; NEE). Positive net ecosystem CO_2_ exchange indicates a net source of carbon to the atmosphere. The error bar for the logged plot average represents the between-plot uncertainty, and the error bar for SAF-05 represents within-plot uncertainty, estimated by propagation of errors of the individually measured components of productivity and respiration.

Both eddy covariance and biometric ground-based estimates independently demonstrated that the logged forest landscape was a net source of carbon to the atmosphere, although there was some discrepancy in the magnitude of this source, with the eddy covariance estimate showing a larger source. The discrepancy between the two methods was expected, due to the inclusion of both moderately and heavily logged plots within the biometric logged forest estimate (Fig. 3), while the eddy covariance tower footprint was almost entirely heavily logged. A direct comparison of the specific biometric plot that falls within the tower footprint (SAF-05) shows net ecosystem CO_2_ exchange estimates that were lower in the plot relative to the eddy covariance methods, but the two had overlapping CIs (Fig. 4). Estimates of both gross primary productivity and ecosystem respiration were approximately equal from the two methods (Fig. 4).

## Discussion

Our data demonstrate that logged tropical forests in Malaysian Borneo represent a substantial net carbon source to the atmosphere for at least a decade after logging. Net ecosystem CO_2_ exchange estimated from eddy covariance was higher (i.e., larger source) than the estimate from the biometric plot within the flux tower footprint (SAF-05); this trend has been observed globally when comparing these methods (21) and can be potentially caused by the plot not fully representing the eddy covariance footprint (22). As both methods show the landscape to be acting as a net source, the main conclusion of this study is not affected by this discrepancy. Logged forest had significantly greater woody productivity than unlogged forests, which is consistent with earlier literature (2, 12–15). However, significantly higher carbon losses from heterotrophic sources in logged forest outpaced the biomass gain and ultimately resulted in a net source of carbon. The magnitude of this net source increased with increasing level of logging intensity. Our results demonstrate critically how focusing on carbon gain from woody biomass accumulation alone (2, 13) does not provide a complete picture of carbon cycling within logged tropical forests, and thus emphasizes the importance of investigating net CO_2_ exchange and complete carbon budgets in logged tropical forests. Estimates of post-disturbance carbon balance recovery from activities such as logging are critically lacking in the literature but need to be included in scaled-up estimates of the net carbon balance of the forest biome (23).

Overall, the lack of significant difference in net primary productivity, gross primary productivity, and autotrophic respiration between logged and unlogged forests indicates similar carbon use efficiency in both forest types. However, the way in which the two forests deliver that carbon use does vary. Trees in logged forests grow faster than unlogged forests, with stem growth rate and recruitment both 50% higher in logged than in unlogged forest (24). Particularly in regrowth stands, above-ground biomass has been shown to accumulate rapidly in the first 20 y following stand-clearing disturbance (25). The growth rate of smaller trees increases as they benefit from increased light availability and decreased competition for resources following logging (17, 26). However, the increased woody production observed here is not due to an increase in total net primary productivity, as this did not differ between logged and unlogged forests (Fig. 2) but was rather due to an allocation shift towards the increased production of woody stem tissue at the expense of canopy leaf tissue (20). This highlights a difference in investment strategies and plant functional traits between trees in logged and unlogged plots, with those in logged plots investing more in their woody structure and gaining height whereas those in unlogged plots invest more heavily in expanding their crown (27). In the tropics, similar shifts between canopy and woody allocation have been reported in naturally regenerating forests (25), contrasting to temperate regions, whereby allocation to canopy remained constant in young and mature stands despite changes in environmental conditions and resource availability (28).

The main difference in the carbon dynamics of logged forest was heterotrophic respiration. We observed major losses from both soil organic matter and deadwood (Fig. 2). Logged forests have large deadwood stocks (29) that originate from abandoned logs, collateral damage during logging, elevated mortality of damaged trees post-logging (30), and the death of first-generation pioneers that colonized the logging gaps (31, 32). The decay of this woody debris ensures that logged forests have elevated rates of deadwood respiration for decades after logging. The elevated soil respiration is likely to originate from the loss of old soil carbon (16), which may also persist for decades after logging (16, 33) although the specific mechanisms are not well understood. It has been previously suggested that forests with larger proportions of their ecosystem carbon stored in their deadwood and soil organic matter will have large net losses of carbon over time whilst all the necromass rots away (34). This emphasizes in turn the necessity to adopt methods such as reduced impact logging for timber extraction, which can minimize damage to vegetation and soil (31) and effectively reduce logging-induced emissions (32). Employing reduced impact logging methods has been shown to retain 23% more forest biomass than conventional methods by reducing tree mortality, which subsequently reduces carbon losses from necromass stocks (34).

Our eddy covariance data show that the landscape was significantly affected by logging activities and allow us to generate a pseudo-chronosequence of events. Between active logging and the initial recovery years, gross primary productivity was unaffected. During logging, herbs, shrubs, and grasses, which quickly colonize new gaps, compensate for the decreased tree stand productivity, as the density of understory vegetation is typically higher in logged forests compared to unlogged areas (35, 36). In the immediate years following a logging event (2 to 3 y recovering), net ecosystem CO_2_ exchange reduces due to a short-term reduction in respiratory processes before productivity begins to increase as smaller trees, trees closer to gaps, and understory vegetation all benefit from increased light availability and reduced competition. Eventually, net ecosystem CO_2_ exchange levels are elevated as the forest continues to regrow (10-y recovering).

Although our data come from one geographic region, the forests we work in share characteristics with tropical forests more widely, and our conclusions therefore have wide relevance. For example, the standardized protocol for the ground-based biometric estimates that we used (37, 38) returned carbon balance estimates for unlogged forests (−0.71 ± 1.23 Mg C ha^−1^ yr^−1^_,_ a net sink) that are comparable to biometric estimates of unlogged forest in other tropical regions, such as 0.8 ± 2.0 Mg C ha^−1^ yr^−1^ (39) and −1.6 ± 4.40 (40) in unlogged Amazonian forests. Moreover, the moderately and heavily logged forests at our study site are not uncommon in tropical and subtropical forests globally. Biomass losses at our study site, [average loss of 50% (20)] are comparable to what has been observed in Africa (41) and Brazil (42) (20 to 72% and 35 to 57%, respectively), and the total basal area at the moderately logged forest plots investigated in this study [20 ± 1.8 m^2^/ha (16)], are comparable to those reported following logging in Uganda, Eastern Africa (~20 m^2^/ha) (43), the Brazilian Amazon (26 m^2^/ha) (44) and Australia (12 to 58 m^2^/ha) (45). We acknowledge, however, that the heavily logged plots we examined represent more of an extreme and unsustainable approach to logging that highlights a worst-case scenario. But such high degradation is—unfortunately—not unique to our study site: low basal areas similar to the heavily logged plots investigated in this study [6.8 ± 1.0 to 14 ± 1.7 m^2^/ha (16)], have been recorded in Indonesia [14 ± 7 and 18 ± 10 m^2^/ha (46)] and in Myanmar [6.2 ± 0.26 m^2^/ha (47)]. Overall, we believe our study site and our data to be broadly representative of the wider logged tropical forest landscape.

## Conclusion

The regrowth of tropical forests recovering from past deforestation and forest degradation is considered to constitute an important carbon sink, but our data challenge this widely held assumption. We have shown a substantial and persistent net carbon source using both eddy covariance and biometric ground-based estimates in logged tropical forests. Despite amplified woody productivity, the net carbon source persisted for at least a decade following logging due to respiratory losses from heterotrophic sources. Although our data come from just one area, the potential implications are serious: the tropical forest carbon sink may be much smaller than previously estimated if recovering forests are a net carbon source. Heterotrophic respiration from soil and from deadwood forms a crucial piece of the puzzle. The impact of logging on these processes may be variable and site-specific, empirical data from the tropics are extremely limited, and models on the fate of soil carbon have large uncertainties, and all of these knowledge gaps now need to be urgently addressed. Given that human-modified forests are so widespread, have high biodiversity value and continue to become an increasingly prevalent part of the tropical forest biome, it is imperative that they are represented accurately within the global carbon budget.

## Materials and Methods

### Study Location.

The study sites were located in lowland, dipterocarp-dominated, humid tropical forests within the states of Sabah and Sarawak in Malaysian Borneo (*SI Appendix,* Table S3). This region is moist tropical and aseasonal, with a daily mean temperature of 26.7°C and annual precipitation of 2,600 to 2,700 mm (48). Sampling was conducted within 11 × 1-ha intensive Global Ecosystem Monitoring (GEM) plots (37, 38), which captured a gradient of logging intensity from heavily logged to unlogged forests, and with one eddy covariance tower in the heavily logged landscape. Logged plots (five plots) were located within the Stability of Altered Forest Ecosystems (SAFE) Project in Kalabakan Forest Reserve (20, 49) on mostly clay soil (*SI Appendix*, Table S3). These plots have been logged two (SAF-03 and SAF-04) or four (SAF-01, SAF-02, SAF-05; eddy covariance tower) times, with the first round of logging taking place in the mid-1970s in all plots, with subsequent rounds during 1990 to 2000s, although the exact logging history of this area is not explicitly documented (29, 50). Over this entire period, approximately 150 to 179 m^3^ ha^−1^ of timber was removed (50) which is similar to the mean extraction rate of 152 m^3^ ha^−1^ across Sabah (51). As this area was set to be converted into an oil palm plantation (49), the usual logging conventions and 60-y rotation period was not followed, which left parts of the area highly degraded (52). The current aboveground carbon stocks in moderately and heavily logged plots are ~70% and ~25%, respectively, of the estimated pre-logging 1970’s aboveground carbon stocks (20). As the data collection for these biometric plots was continuously measured over multiple years (2011 to 2017), the heavily logged plot estimates represent carbon dynamics at ~10 y recovery and moderately logged at ~20 y recovery. Parts of the area, including >90% of the flux tower footprint, but not the biometric plots, were salvage logged in 2015. Old-growth plots were located within Maliau Basin Conservation Area (Sabah; two plots), Danum Valley Conservation Area (Sabah; two plots), and Lambir Hills National Park (Sarawak; two plots). Plots within Maliau Basin Conservation Area, Danum Valley Conservation Area and one plot within Lambir Hills were located on clay soils, and the other Lambir Hills plot on sandy loam (*SI Appendix*, Table S3). For more detailed site and plot descriptions, including species composition, soil properties, logging history, and a map, see ref. 20.

### Eddy Covariance Data Collection.

This study used data from a 50-m scaffolded eddy covariance tower (4° 43.091′ N, 117° 36.246′ E) installed in 2011, which has recorded both meteorological data (53) and eddy flux (previously unpublished) from August 2012 to 2019. Details of the measuring system and post-processing steps are available in *SI Appendix*, S1. Data were collected over three measuring periods: in 2012 to 2013, which captured the four-times logged ecosystem ~10 y after its previous round of logging, in 2015 during a new round of active salvage logging, and in 2017 to 2018 when the ecosystem was recovering 2 to 3 y after the salvage logging. The salvage logging in 2015 removed approximately 75% of tree stand basal area, through direct timber extraction and collateral damage.

Missing data is a common problem in long term eddy covariance experiments due to mechanical failure, system maintenance, power failure, lightning strikes, and low wind speed. Due to this, between 2012 and 2018 the tower recorded data for only 51% of this time. This resulted in 455 days being sampled over this period, with 65 days during 10-y recovery, 100 days during active salvage logging and 290 days during the 2 to 3 y recovery from active salvage logging. Hence, due to the lack of continuous data per annum, it was most appropriate to employ daily estimates for this method, as these estimates are more robust and avoid large periods of continuous gap-filled data, particularly as the climate is aseasonal. Further quality control included the application of a friction velocity (u*) threshold and gap filling. A mean threshold of u* >0.29 m s^−1^ was applied to the dataset, as established using the package “REddyProc” [v.1.2; (54)] in R (v.4.0.2; R Core Team, 2019) based on the Moving Point Method (55). The remaining data were gap-filled using marginal distribution sampling (MDS) (56) using the R package “REddyProc” (54). Of the final dataset, 29.5% was original observed fluxes, and 70.5% were gap-filled (only days with observations were gap filled). Data were partitioned into gross primary productivity and ecosystem respiration using a daytime light response with VPD limitation (57) with a VPD_0_ threshold of 10 hPa (58), fitted to 7-day moving windows (*SI Appendix*, S2). Gross primary productivity was subsequently calculated as gross primary productivity = ecosystem respiration - net ecosystem CO_2_ exchange. We used the root sum of squares to calculate an uncertainty estimate for net ecosystem CO_2_ exchange, including random uncertainty and gap-filling uncertainty. For this, the distribution of various sources of random error are propagated (e.g., u*, wind speed, air density, momentum flux) with gap-filling uncertainty (propagation of the SD for each gap filled value produced by the ReddyProc package), and with the SD of all observed values (*SI Appendix*, S3). Random error contributed 98% of the total estimated uncertainty for net ecosystem CO_2_ exchange and resulted in a total error estimate of 17% for net ecosystem CO_2_ exchange. For ecosystem respiration and gross primary productivity, we used ±1 SD of the daily estimates to represent the error, which was 23% for ecosystem respiration and 16% for gross primary productivity. Wilcox statistical test was applied to determine the difference in net ecosystem CO_2_ exchange, gross primary productivity, and ecosystem respiration between measuring periods using R (v.4.0.2; R Core Team, 2019).

### Biometric Estimates.

We quantified components of net primary productivity, ecosystem respiration including heterotrophic and autotrophic respiration, gross primary productivity, and subsequently net ecosystem CO_2_ exchange, as the difference between ecosystem respiration and gross primary productivity. Methods employed for biometric-ground based sampling are described in refs. 16, 20, 37, and 38. Biometric data collection methods are summarized in the *SI Appendix*, Table S4. Total measured net primary productivity included woody, canopy, and fine root productivity, described in detail in ref. 20. Briefly, stem and coarse root woody net primary productivity was estimated from repeated tree censuses and allometric equations, canopy net primary productivity was derived from litterfall traps, whereby fine litterfall of <2 cm in diameter was used as a proxy for canopy production, and fine root net primary productivity was estimated using root ingrowth cores. Net primary productivity data were collected during 2011 to 2017 for SAFE, Maliau and Danum (Sabah) plots over a minimum of 24 months at each plot and each plot was subject to at least two tree censuses. Plots in Lambir (Sarawak) were censused every 5 y from 1992 to 2008 and net primary productivity data were collected between 2008 and 2010 over 15 months. Net primary productivity data used in this study has been already published elsewhere (20, 59).

Soil, stem, leaf, and deadwood respiration was measured. Total soil respiration was partitioned into autotrophic (root respiration) and heterotrophic (litter, mycorrhiza, and soil organic matter respiration) using collars that selectively excluded each component [for full details of the soil respiration methods see ref. 16]. Stem respiration was measured from 30 to 40 living trees per plot and scaled to the surface area of the 1-ha plot by estimating the total stem area using tree census data and an allometric equation between tree diameter and stem surface area (60). Respiration of deadwood was measured on 25 deadwood pieces per plot and scaled to the plot level by estimating the total deadwood surface area in the plot using data on deadwood inventory where all deadwood pieces ≥10 cm diameter were measured. As the stem respiration and deadwood respiration data have not been previously published elsewhere, the methods are described in detail in *SI Appendix*, S4 and S5. Leaf respiration was measured during one campaign within each plot during 2015 for SAFE, Maliau and Danum. For each plot, species were ranked by their basal area and species which contributed to 70% of total basal area of the plot were sampled (27). Mean dark respiration of sun and shade leaves were multiplied by their estimated fractions in each plot and then multiplied by the leaf area index of the plot (16, 61). In Lambir, leaf respiration estimates were obtained from a previous study (62) and scaled to the plot level by multiplying by the leaf area of the plot. In all plots, an inhibition correction factor of 0.67 was used to account for the daytime light inhibition of leaf dark respiration (40).

Heterotrophic respiration was quantified as the sum of litter, mycorrhiza, soil organic matter and deadwood respiration. Autotrophic respiration was the sum of woody, fine root, and leaf respiration. The following equations were then applied, whereby ecosystem respiration = heterotrophic respiration + autotrophic respiration_,_ and gross primary productivity = net primary productivity + autotrophic respiration and subsequently net ecosystem CO_2_ exchange = ecosystem respiration – gross primary productivity (or, equivalently, net ecosystem CO_2_ exchange = heterotrophic respiration – net primary productivity). We adopt the sign convention that negative values of net ecosystem CO_2_ exchange indicate ecosystem uptake of CO_2_ and positive values a source of CO_2_ from the ecosystem to the atmosphere. Statistical analysis for comparing the carbon cycle components between logged and unlogged forest types was conducted in R (v.4.0.2; R Core Team, 2019), using t test and Wilcox rank-sum test.

The plot-level (n = 11 plots) estimates of the complete carbon budget components are available at https://doi.org/10.5281/zenodo.7307449 and eddy covariance data flux data, both raw and daily estimates are available at https://doi.org/10.5281/zenodo.7307447, with associated microclimate data at https://doi.org/10.5281/zenodo.3888375.

## Supplementary Material

Appendix 01 (PDF)

## Data Availability

[field datasets; microclimate data] data have been deposited in [Zenodo] (https://doi.org/10.5281/zenodo.7307449, https://doi.org/10.5281/zenodo.7307447, and https://doi.org/10.5281/zenodo.3888375). Previously published data were used for this work [L. K. Kho et al. (59), T. Riutta et al. (16), and T. Riutta, et al. (20)].
