# Supplementary material for: Tropical forests post-logging are a persistent net carbon source to the atmosphere
Source: Proc Natl Acad Sci U S A. 2023 Jan 9;120(3):e2214462120. doi: 10.1073/pnas.2214462120 (PMC9934015; doi:10.1073/pnas.2214462120)
Supplement: Supplementary file 1 — Appendix 01 (PDF) [file pnas.2214462120.sapp.pdf]

Supporting Information for
Tropical forests post-logging are a persistent net carbon source to the atmosphere

Maria B. Mills, Yadvinder Malhi, Robert M. Ewers, Lip K. Kho, Yit A. Teh, Sabine Both, David F. R. P Burslem, Noreen Majalap, Reuben Nilus, Walter Huaraca Huasco, Rudi Cruz, Milenka M. Pillco, Edgar C. Turner, Glen Reynolds, and Terhi Riutta

Maria B Mills
Email: mbm19@leicester.ac.uk

This PDF file includes:

Supporting text S1 – S5
Tables S1 to S4
Figure S1
SI References

Supporting Information Text (S1 – S5)

S1

Measuring system. The measuring system consists of a semi-open path infrared gas analyser LI-7200 (LI-COR, USA), and a CSAT3 Sonic Anemometer (Campbell Scientific, USA) at a measuring height of 52 m over a canopy height of ~25 m. The above-canopy measurement system consists of temperature and relative humidity (RH) probes (HMP115, Vaisala, Finland) with an unaspirated radiation shield (MET21, Campbell Scientific, USA), and photosynthetically active radiation (PAR) with a Quantum Sensor (SKP215, Skye, UK). Below the canopy, volumetric water content is measured with a water content reflectometer at depths of 5 cm, 10 cm, and 20 cm (CS616, Campbell Scientific, USA). Above- and below-canopy microclimate is recorded with automatic dataloggers (CR1000, Campbell Scientific, USA). Data were recorded at a frequency of 20 Hz that was treated using the post-processing software EddyPro® (v.7.0.6; www.licor.com/eddypro) to compute fluxes for each 30-minute averaging period. To treat the raw fluxes, primary data processing steps were applied, including spike removal (1), coordinate rotation, block averaging detrending of CO_2, H_2O and sonic temperature, time lag compensation using covariance maximization detection method, random uncertainty estimation (2), computation of turbulent fluxes and mean fluxes, spectral corrections (3) using correction of low-pass filtering effects, planar fit rotation (4) and quality flagging policy (5). Net ecosystem CO_2 exchange (NEE) was calculated by adding the estimated CO_2 storage flux to the observed CO_2 flux. Secondary data processing was applied to remove spurious data, often associated with non-stationary meteorological conditions (6). Quality flags 4 and 5 (5) for CO_2 flux were removed and further flux spike removal was applied (6).

S2

Gap filling and flux partitioning procedure. Data were gap-filled using marginal distribution sampling (7) using the R package "REddyProc" (8). It was necessary to further quality control this gap-filled data as it introduced spurious values into the dataset. To quality control, the average value and 90% confidence interval for each 30-minute interval were calculated per measuring period (namely, 10-years recovering, active salvage logging, and 2-3 years recovering) to create an idealized diurnal pattern based on observed values only. Gap-filled values outside of this 90% confidence interval range were replaced with the corresponding upper or lower confidence interval limit.

Data were partitioned into gross primary productivity (GPP) and ecosystem respiration (R_{eco}) by fitting a light response curve with vapor pressure deficit (VPD) limitation (9) to the daytime data (observed values between 08:00 – 19:00, no gap-filled data was included), using 7-day moving windows (Equation 1). The model intercept was used as an estimate of R_{eco}.

Equation 1:

$$NEE = VPD > VPD_0, -\beta \exp\left(-k(VPD - VPD_0\right)\left(\frac{1 - \exp(-\alpha PAR)}{\beta \exp\left(-k(VPD - VPD_0)\right)}\right) + \gamma\,,$$

$$VPD \leq VPD_0, -(\beta + \gamma)(1 - exp\left(\frac{\alpha PAR}{\beta + \gamma}\right) + \gamma)$$

Whereby, β is the maximum CO_2 uptake rate of the canopy at light saturation**,** α is canopy light utilization which represents the initial slope of the light–response curve, γ is ecosystem respiration and PAR is photosynthetically active radiation. Parameter *k* was set to a constant value, which was derived from a separate VPD response curve. The VPD response curve was fitted to the whole dataset but using only light-saturated (photosynthetically active radiation, PAR>1200) and VPD limited (>VPD_0) conditions, assuming a VPD_0 threshold of 10hPa (10). A constant value for α was applied which was the slope of the linear regression between NEE and PAR under low light conditions (<200 µmol) (11), which was executed per measuring period (10 years recovering, active salvage logging and 2-3 years recovering). Example code for partitioning net ecosystem CO_2 exchange is available at https://github.com/mbmills19/EC-P.git

Days with large standard errors for R_{eco} (> ± 5 µmol m^{-2} s^{-1}) were deemed as bad quality and removed from the dataset. Estimated R_{eco} from this daytime partitioning method was used to replace night-time values of NEE (18:30 – 06:30). For the final dataset for this study, we used only days that had four or more observed half-hourly values of NEE. Because the study site has an aseasonal climate, we decided on this approach as opposed to using large amounts of gap-filled data to create a continuous dataset, to reduce the possibility of bias and provide more robust estimates on a daily scale. Due to the large gaps in our dataset, which is a common problem associated with the eddy covariance method, adopting large periods of completely gap-filled data would lead to more gap-filled than observed data. Of the final dataset used in this study, 29.5% of the half-hourly values are original observed fluxes, and 70.5% gap-filled. Of the 455 days remaining after all filtering processes were applied, 65 days were during the 10-years recovery phase (2012-2013), 100 during the active salvage logging (2015) and 290 during the 2-3 years recovery from active salvage logging phase (2017-2018).

S3

Random uncertainty of NEE. We used the root sum of squares to estimate the random uncertainty of NEE by propagating the standard error of the distribution of the various sources of errors using empirical data. The sources of random error include global radiation, frictional velocity (u*), momentum flux, wind speed, air density, turbulent kinetic energy, relative humidity, evapotranspiration, air temperature and random uncertainty of CO_2 flux (as

generated in EddyPro®; v.7.0.6; www.licor.com/eddypro). These errors were propagated with standard error of NEE and gap-filling uncertainty, which is derived from the standard deviation of each gap-filled value as generated by REddyProc. Propagating from all these distributions of errors resulted in an NEE uncertainty estimate of 2.06 Mg C ha^{-1} yr^{-1}. The random uncertainty of NEE was ~98% of the total uncertainty estimate. For R_{eco} and GPP, we used the standard deviation of the dataset to represent the error, and we propagated the random uncertainty of NEE with the standard error of NEE, GPP and R_{eco} to calculate the 95% confidence interval of each estimate.

S4

Respiration estimates from woody stems. Stem respiration was measured with a static chamber technique using standardized techniques from the Global Ecosystem Monitoring (GEM) protocol (12, 13). Sampling was conducted monthly, with intermittent gaps due to issues with access, staffing and equipment, between 2011 – 2019 for SAFE and Maliau plots (Sabah), 2015 – 2019 for Danum (Sabah), and 2008 – 2009 Lambir (Sarawak). Approximately 40-50 stems per plot were sampled, of various sizes and species and distributed evenly around the plot. A 5 cm high PVC collar with a 10.6 cm internal diameter was installed on each stem at 1.1 m height with silicone sealant. Respiration from the stem was measured with an IRGA and soil respiration chamber (EGM-4 and SCR-1, PP Systems, USA). A custom ring adapter of 11 cm diameter and 3.5 cm height was fitted to the chamber to match the diameter of the collars and enable an air-tight seal to avoid leakage (7). Before each measurement, the chamber was flushed, and the collar fanned to remove stagnant air. Any mosses, epiphytes or insect nests were also removed from the collar before each measurement. The chamber was then placed onto the collar and CO_2 efflux was measured for 120 seconds. The flux was calculated as the linear change in CO_2 concentration (ppm) in the chamber headspace (total volume of the chamber, adapter piece and respiration collar) and corrected for air temperature. Data were subject to quality control, removing spurious data which were considered as mechanical issues, human error or outside of logical bounds. Data from 2016 was removed from the dataset as the region experienced a strong El Niño event during this year. For each plot, outliers outside 1.5 of the interquartile range above the upper quartile or below the lower quartile were removed. Each plot was manually investigated for further outliers. Raw data (ppm) were converted to estimate CO_2 efflux per unit of stem surface area per month (g C m^{-2} $month^{-1}$) and were corrected to 25°C using a Q10 of 2.0 (14). Plot level stem respiration was estimated by taking an average for each plot, based on monthly estimates of stem respiration at each plot. This was to ensure months were weighted evenly and to avoid sampled trees being weighted unequally. This estimate was scaled to the 1-ha plots by estimating the total stem surface area of each plot using the tree census data (15) and an allometric equation between stem diameter and stem surface area (16), and converted to Mg C ha^{-1} yr^{-1}.

S5

Respiration estimates from deadwood. Measurements and data processing of the respiration from deadwood followed the same protocol as that of standing trees, except that the collars were installed on 25 fallen deadwood pieces ≥ 10 cm diameter per plot. For each subplot, the deadwood piece nearest to the subplot center was chosen. For scaling the deadwood measurements to the plot level, a full inventory of all deadwood pieces was carried out in each plot, where all deadwood pieces ≥ 10 cm diameter per plot were measured (total length and diameter at each end). The surface area of each deadwood piece was calculated as the surface area of a cut cone and summed for each plot. This was corrected by 0.66 to account for the surface area of fallen deadwood which is against the ground and assumed to be respiring at a lower rate. Estimates of deadwood respiration per unit surface area were then scaled by the estimated plot-level deadwood surface area to represent respiration from deadwood in Mg C ha^{-1} yr^{-1}. In the Lambir plots (LAM-05 and LAM-06), deadwood respiration was not directly measured. Instead, it was estimated from the measured coarse woody debris inputs (17).

Table S1: Eddy covariance estimates per measuring period

Eddy covariance estimates of net ecosystem CO_2 exchange (NEE), gross primary productivity (GPP) and ecosystem respiration (R_{eco}) during the three periods of data collection over a heavily logged forest landscape within the SAFE project, nearby to SAF-05 plot. Different letters after the component estimates indicate significant differences (Wilcoxon signed-rank test, $p<0.05$) between periods. The eddy covariance site was 10-years recovering from four rounds of logging before being salvage logged in 2015 and then entered a new period of recovery, of which was all captured by the eddy covariance flux tower. Photographs of the landscape during these data collection periods are provided within SI Appendix (Fig. S1).

Period	NEE<br>(g C m^{-2} d^{-1})	GPP<br>(g C m^{-2} d^{-1})	R_{eco}<br>(g C m^{-2} d^{-1})
2012-2013: 10-years recovering[1]	5.4 ± 1.99 (A)	9.87 ± 1.66 (A)	15.28 ± 3.49 (A)
2015: Active salvage logging[2]	4.41 + 1.20 (B)	8.92 ± 1.26 (B)	13.33 ± 2.18 (B)
2017-2018: 2-3 years recovering[3]	2.53 ± 1.22 (C)	8.52 ± 1.28 (B)	11.11 ± 2.7 (C)

[1] At this point, the landscape was ~ten years recovering from the latest round of logging, having gone through four rounds of selective logging, first round in 1970's and the subsequent rounds during 1990s-2000s. In 2012 it had approximately 30% of its estimated 1970's pre-logging biomass remaining (Riutta et al. 2018).

[2] The landscape (>90% of the footprint) was salvaged logged during March - June 2015, with the exception of the biometric SAF-05 plot (1-ha), some steep slopes and riparian buffers. This round of logging was more extreme than the previous rounds, as the landscape was earmarked for conversion to oil palm, and removed 75% of the stand basal area.

[3] The landscape was recovering from the 2015 salvage logging.

Table S2: Carbon budget components for biometric plots

Components of the complete carbon budget of both logged (n = 5) and unlogged (n = 6) biometric plots with ± 1 standard error. Positive values of net ecosystem exchange indicate a net source of CO_2 to the atmosphere. Previously published data has been numerically denoted, with source acknowledges on the final rows.

Component		Logged forest ($Mg\ C\ ha^{-1}\ yr^{-1}$)	Old-growth forest ($Mg\ C\ ha^{-1}\ yr^{-1}$)
**Net Primary Productivity (NPP)**[1]	NPP_{Woody}	9.77 ± 0.81	5.54 ± 0.62
	NPP_{Stem}	7.13 ± 0.63	3.95 ± 0.54
	NPP_{Branch}	0.73 ± 0.17	0.64 ± 0.12
	$NPP_{Coarseroots}$	1.91 ± 0.26	0.95 ± 0.14
	NPP_{Canopy}	3.77 ± 0.19	6.88 ± 0.40
	NPP_{Leaf}	3.01 ± 0.15	5.11 ± 0.26
	NPP_{Twig}	0.31 ± 0.06	0.85 ± 0.15
	$NPP_{Reproductive}$	0.06 ± 0.03	0.21 ± 0.08
	$NPP_{Miscellanous}$	0.09 ± 0.01	0.30 ± 0.10
	$NPP_{Herbivory}$	0.30 ± 0.02	0.40 ± 0.07
	$NPP_{Fineroots}$	1.54 ± 0.32	1.21 ± 0.20
	$NPP_{Mycorrhiza}$	0.85 ± 0.36	1.40 ± 0.40
	**Total NPP**	**15.93 ± 1.28**	**15.03 ± 0.75**
**Ecosystem Respiration (R_{eco})**	**R_h**	**19.77 ± 1.51**	**14.32 ± 0.64**
	R_{SOM}[2]	7.82 ± 0.32	6.32 ± 0.39
	$R_{Mycorrhiza}$[2]	0.85 ± 0.36	1.40 ± 0.40
	R_{Litter}[2]	3.10± 0.34	2.35 ± 0.26
	$R_{Deadwood}$	8.01 ± 1.37	4.25 ± 0.53
	**R_a**	**20.77 ± 1.29**	**20.03 ± 0.53**
	R_{Stem}	6.69 ± 1.06	6.18 ± 0.48
	R_{Leaf}[3]	9.73 ± 0.40	9.76 ± 0.46
	$R_{Fineroots}$[2]	2.61 ± 0.65	2.61 ± 0.09
	$R_{Coarseroots}$[2]	1.77 ± 0.28	1.48 ± 0.11
**Ecosystem Respiration (R_{eco}) = R_h + R_a**		**40.54 ± 2.03**	**34.36 ± 0.96**
**Gross Primary Productivity (GPP) = NPP + R_a**		**36.70 ± 2.28**	**35.06 ± 1.13**
**Net Ecosystem Exchange (NEE) = R_{eco} - GPP**		**3.84 ± 1.13**	**-0.71 ± 1.23**

[1] *Previously published in Riutta et al. (2018) and Kho et al. (2013)*
[2] *Previously published in Riutta et al. (2021)*
[3] *Previously published in Both et al. (2019) (Leaf-scale estimates only)*

Table S3: Study plot characteristics

Characteristics of study plots including plot code as it appears in the forestplot.net database, site and coordinates, logging intensity, aboveground biomass (AGB) in Mg C ha^{-1} yr^{-1} ± 1 standard error, soil type, topography, and the three most abundant genera in the plot. Further information on characteristics is available in Riutta et al. (2018, 2021)

Plot Code	Site and coordinates	Logging intensity	AGB (Mg C ha^{-1})	Soil type; topography	Most abundant genera
MLA-01	Maliau Basin Conservation Area, Sabah (4.747°, 1176.970°)	Old-growth	278 ± 31	Clay; undulating	*Shorea, Dryobalanops, Eusideroxylon*
MLA-02	Maliau Basin Conservation Area, Sabah (4.754°, 1176.950°)	Old-growth	284 ± 26	Clay; moderate slope	*Parashorea, Mallotus, Shorea*
DAN-04	Danum Valley Conservation Area, Sabah (4.951°, 117.796°)	Old-growth	206 ± 27	Clay; steep slope	*Shorea, Cleistanthus, Parashorea*
DAN-05	Danum Valley Conservation Area, Sabah (4.953°, 117.793°)	Old-growth	191 ± 35	Clay; flat	*Diospyros, Aglaia, Shorea*
LAM-06	Lambir Hills National Park Sarawak (4.183°, 114.002°)	Old-growth	254 ± 19	Sandy loam; undulating with steep slopes	*Shorea, Elateriospermum, Dryobalanops*
LAM-07	Lambir Hills National Park Sarawak (4.188°, 114.019°)	Old-growth	216 ± 49	Clay; valley	*Koilodepas, Shorea, Dryobalanops*
SAF-03	SAFE Project, Sabah (4.691°, 117.588°)	Moderately logged	97 ± 13	Clay; steep slope	*Macaranga, Shorea, Litsea*
SAF-04	SAFE Project, Sabah (4.765°, 117.700°)	Moderately logged	111 ± 11	Partly sandy loam, partly clay; flat	*Syzygium, Shorea, Vatica*
SAF-01	SAFE Project, Sabah (4.732°, 117.619°)	Heavily logged	31 ± 5	Clay; mostly flat with a moderate slope on one edge	*Macaranga, Shorea, Dendrocnide*
SAF-02	SAFE Project, Sabah (4.739°, 117.617°)	Heavily logged	50 ± 9	Clay; undulating	*Macaranga, Shorea, Mallotus*
SAF-05	SAFE Project, Sabah (4.716°, 117.609°)	Heavily logged	56 ± 10	Clay; undulating	*Dendrocnide, Macaranga, Parashorea*

Table S4: Summary of methods

Summary of methods for intensive carbon monitoring of net primary productivity (NPP) and ecosystem respiration (R_{eco}) including autotrophic (R_a) and heterotrophic respiration (R_h). Details of previous studies where these results are published are cited.

Component			Description
**Net primary productivity (NPP)**		Woody NPP (NPP_{Woody})	Sum of stems, coarse roots, and branches which is estimated from repeated tree censuses and allometric equations (15)
		Canopy NPP (NPP_{Canopy})	Sum of leaves, twigs, and reproductive parts derived from litterfall traps (15)
		Fine Root NPP ($NPP_{Fineroots}$)	Estimated using root ingrowth cores (15)
**Ecosystem Respiration (R_{eco})**		Total soil CO_2 efflux (R_{Soil})	Total soil respiration was estimated from monthly measurements of soil CO_2 fluxes using permanent collars and the static chamber method. Total soil respiration was partitioned into **autotrophic respiration** (root respiration) and **heterotrophic respiration** (litter, mycorrhiza, and soil organic matter respiration) using collars that selectively excluded each component (18)
	**Heterotrophic respiration (R_h)**	Respiration from soil organic matter (R_{SOM})	Derived from total soil respiration (18)
		Respiration from mycorrhiza ($R_{Mycorrhiza}$)	Derived from total soil respiration (18)
		Respiration from (R_{Litter})	Derived from total soil respiration (18)
		Respiration from deadwood ($R_{Deadwood}$)	Respiration from deadwood is measured using static chamber technique using permanent fixed collars. Respiration estimates are then upscaled to the deadwood surface area of the plot. The surface area of the fallen deadwood biomass is corrected to 0.66 to account for the surface area against the ground that is respiring less
	**Autotrophic respiration (R_a)**	Coarse root respiration ($R_{Coarseroots}$)	Derived from total soil respiration (18)

Fine root respiration ($R_{Fineroots}$)	Derived from total soil respiration (18)
Leaf and canopy respiration (R_{Leaf})	Measured during one campaign within each plot. If both shaded and fully lit branches were available, both were sampled. Leaf-level measurements were scaled to plot level. Mean dark respiration of sun and shade leaves were multiplied by their estimated fractions in each plot and then multiplied by the leaf area index of the plot. An inhibition correction factor of 0.67 for the daytime hours was used to account for the daytime light inhibition of leaf dark respiration (17, 19)
Stem respiration (R_{Stem})	Respiration from living stems was measured using static chamber technique from 40 - 50 trees per plot, evenly distributed around the plot. Estimates are scaled to the surface area of the 1-ha plot by estimating the total stem surface area using tree census data and allometric equations.

Fig. S1: Photographs of the data collection periods from the top of the eddy covariance tower

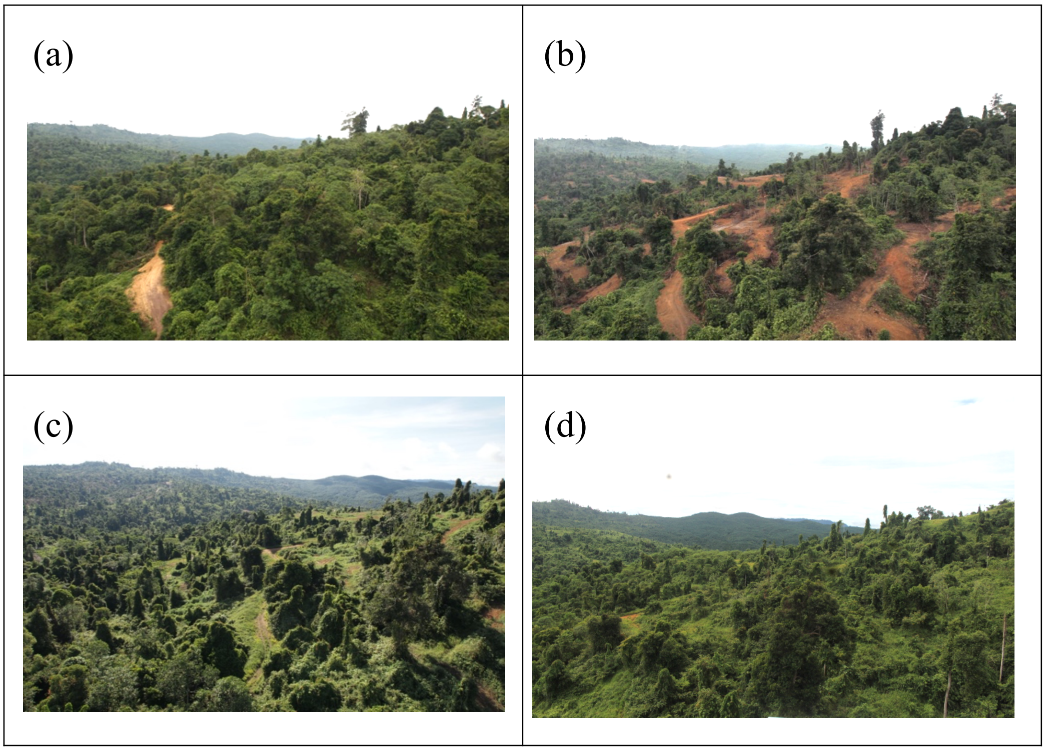

From 2015 – 17, biweekly photographs were taken from the top (52 m) of the eddy covariance flux tower at SAFE project in north, south, east, and west aspects to document the change in landscape throughout the salvage logging. We have selected multiple photos from through this period to illustrate our data collection periods, whereby (a) shows the landscape in February 2015, indicative of the ~10 year recovery period (as no photos were taken in 2012 – 2013, but we can assume it would be similar), (b) during the peak of the active salvage logging event in May 2015, (c) when the landscape had greened over towards the end of the active salvage logging period in November 2015 and (d) in October 2017 during the 2-3 year recovery period.

SI References:

1. D. Vickers, L. Mahrt, Quality control and flux sampling problems for tower and aircraft data. *J Atmos Ocean Technol* **14**, 512–526 (1997).
2. P. L. Finkelstein, P. F. Sims, Sampling error in eddy correlation flux measurements. *Journal of Geophysical Research Atmospheres* **106**, 3503–3509 (2001).
3. J. B. Moncrieff, *et al.*, A system to measure surface fluxes of momentum, sensible heat, water vapour and carbon dioxide. *J Hydrol (Amst)* **188–189**, 589–611 (1997).
4. J. M. Wilczak, S. P. Oncley, S. A. Stage, Sonic anemometer tilt correction algorithms. *Boundary Layer Meteorol* **99**, 127–150 (2001).
5. M. Göckede, T. Markkanen, C. B. Hasager, T. Foken, Update of a footprint-based approach for the characterisation of complex measurement sites. *Boundary Layer Meteorol* **118**, 635–655 (2006).
6. M. V Thomas, *et al.*, Carbon dioxide fluxes over an ancient broadleaved deciduous woodland in southern England. *Biogeosciences* **8**, 1595–1613 (2011).
7. M. Reichstein, *et al.*, On the separation of net ecosystem exchange into assimilation and ecosystem respiration: review and improved algorithm. *Glob Chang Biol* **11**, 1424–1439 (2005).
8. T. Wutzler, *et al.*, Basic and extensible post-processing of eddy covariance flux data with REddyProc. *Biogeosciences* **15**, 5015–5030 (2018).
9. G. Lasslop, *et al.*, Separation of net ecosystem exchange into assimilation and respiration using a light response curve approach: critical issues and global evaluation. *Glob Chang Biol* **16**, 187–208 (2010).
10. Ch. Körner, “Leaf diffusive conductances in the major vegetation types of the globe” in *Ecophysiology of Photosynthesis*, 100th Ed., E. D. Schulze, M. M. M.Caldwell, Eds. (Springer Study Edition, 1995), pp. 463–490.
11. J. Xu, *et al.*, A general non-rectangular hyperbola equation for photosynthetic light response curve of rice at various leaf ages. *Sci Rep* **9**, 1–8 (2019).
12. Y. Malhi, *et al.*, The Global Ecosystems Monitoring network: Monitoring ecosystem productivity and carbon cycling across the tropics. *Biol Conserv* **253** (2021).
13. T. Marthews, *et al.*, “Measuring Tropical Forest Carbon Allocation and Cycling: A RAINFOR-GEM Field Manual for Intensive Census Plots” (2014) (April 21, 2021).
14. M. A. Cavaleri, S. F. Oberbauer, M. G. Ryan, Wood CO2 efflux in a primary tropical rain forest. *Glob Chang Biol* **12**, 2442–2458 (2006).
15. T. Riutta, *et al.*, Logging disturbance shifts net primary productivity and its allocation in Bornean tropical forests. *Glob Chang Biol* **24**, 2913–2928 (2018).
16. J. Q. Chambers, *et al.*, Respiration from a tropical forest ecosystem: partitioning of sources and low carbon use efficiency. *Ecological Applications* **14**, 72–88 (2004).
17. Y. Malhi, *et al.*, Comprehensive assessment of carbon productivity, allocation and storage in three Amazonian forests. *Glob Chang Biol* **15**, 1255–1274 (2009).
18. T. Riutta, *et al.*, Major and persistent shifts in below-ground carbon dynamics and soil respiration following logging in tropical forests. *Glob Chang Biol* **27**, 1–16 (2021).
19. S. Both, *et al.*, Logging and soil nutrients independently explain plant trait expression in tropical forests. *New Phytologist* **221**, 1853–1865 (2019).
